# The Ethanol Extract of *Holotrichia diomphalia* Larvae, Containing Fatty acids and Amino acids, Exerts Anti-Asthmatic Effects through Inhibition of the GATA-3/Th2 Signaling Pathway in Asthmatic Mice

**DOI:** 10.3390/molecules24050852

**Published:** 2019-02-28

**Authors:** Jung-Hee Hong, Seung-Hyung Kim, Young-Cheol Lee

**Affiliations:** 1 Department of Herbology, College of Korean Medicine, Sangji University, Wonju 26339, Korea; anifam@hanmail.net; 2 Institute of Traditional Medicine & Bioscience, Daejeon University, Daejeon 34520, Korea; sksh518@dju.ac.kr

**Keywords:** *Holotrichia diomphalia* larvae, asthma, IL-5, IL-13, GATA-3

## Abstract

*Holotrichia diomphalia* larvae (HD), a natural product from an insect resource, possesses many pharmacological properties, including anticoagulant, antitumor, anti-inflammatory, and analgesic activity. The major bioactive ingredients include oleic acid, palmitic acid, palmitoleic acid, linoleic acid, proline, and glutamic acid. Although HD is associated with immunoregulatory activities in allergic diseases, the therapeutic mechanisms of the action of HD in allergic diseases have not been investigated. The aim of this study was to evaluate the anti-asthmatic potential of HD in an ovalbumin (OVA)-induced mouse model of allergic asthma. Moreover, the anti-inflammatory potential of HD was examined to identify a plausible mechanism of action of HD in vitro. HD strongly reduced goblet cell hyperplasia, eosinophil infiltration, and reactive oxygen species (ROS), which reduced airway hyperresponsiveness (AHR), inflammation, and the expression of Th2 cytokines (IL-5 and IL-13) in bronchoalveolar lavage fluid (BALF). The expression of IL-5, IL-4, eotaxin-2, lysyl oxidase-like 2 (loxl2), and GATA-binding protein 3 (GATA-3) was attenuated in the lungs. In an in vitro assay, HD exerted immunomodulatory effects through the suppression of Th2 cytokines (IL-5, IL-13), IL-17, and tumor necrosis factor (TNF)-α production through downregulation of GATA-3 expression in EL-4 T cells. These findings suggest that the anti-asthmatic activity of HD may occur through the suppression of Th2 cytokines and total Immunoglobulin E (IgE) production by inhibition of the GATA-3 transcription pathway. Our results suggest that HD may be a potential alternative therapy, or a novel therapeutic traditional medicine, for the treatment of allergic asthma.

## 1. Introduction

Numerous natural products and their major compounds are used as the traditional medicines in many countries. Among them, insects have been relatively well explored as potential traditional sources of natural antioxidants and anti-inflammatory materials. *Holotrichia diomphalia* larvae (HD) have been used traditionally in Korea and China for the treatment of inflammatory diseases, chronic asthma, edema, liver cirrhosis, furuncle, and apoplexy [1,2]. Recently, antibacterial proteins have been isolated from HD [3]. In HD-treated macrophages, the levels of hydrogen peroxide (H_2_O_2_), IL-1, IL-6, and IL-10 were very low [2]. Previous results showed that HD can diminish the extent of hepatocellular damage and may be a potential antifibrotic agent for the treatment of liver fibrosis and cirrhosis [4]. Moreover, a crude extract of HD exerted anticoagulant activity [5].

Recent studies have showed that the protein content of HD was 33.4–44.4% and that several different types of amino acid were present. Of these, seven amino acids were essential to human life. The content of glutamic acid was the highest out of seventeen amino acids [6]. Twenty-two components were identified in the petroleum ether extract of HD. The major components were oleic acid, palmitic acid, and palmitoleic acid [7]. Our results were similar to those reported previously. A recent report has indicated that fatty acids can exert a beneficial effect on lung disease, including some types of asthma [8], and that a combination of fatty acids was a potential therapeutic material for cutaneous inflammatory disorders and allergies [9]. As HD can be used as an effective anti-inflammatory material, we hypothesized that HD could inhibit airway inflammation.

Allergic asthma is a complex inflammatory disease, characterized by Th2-dominant lung inflammation, AHR, remodeling, epithelial cell hyperplasia, and subepithelial fibrosis [10]. Activated Th2 cells induce eosinophil infiltration, which exacerbates airway inflammation and allergic responses in the lungs [11]. Th2 cells and Th2 cytokines induce goblet cell hyperplasia and mucus hypersecretion, which, in turn, induce respiratory obstruction and oxidative responses that contribute to lung damage. Therefore, attenuation of the inappropriate activation of Th2 cells is crucial for the treatment of asthma [12]. Eosinophils have been associated with Th2 cytokines in allergic asthma; Th2 cytokines (IL-5 and IL-13) have been shown to induce eosinophilic inflammation of the airway [13]. IL-4 and IL-13 are known to stimulate the secretion of IgE from B cells, and were found to obstruct the airway and alveolar epithelial barrier, which may amount to airway inflammation [14,15]. T-bet is an important transcription factor, thought to initiate Th1 development, whereas GATA-3 plays a crucial role in the development of the Th2 cells [16]. GATA-3 transcription factor has been shown to increase the expression of Th2 cytokines (IL-4, IL-5, and IL-13) [17].

Signal transducer and activator of transcription (STAT) 6 has been identified as a mediator of asthma, inducing the expression of the Th2 master regulator GATA-3, which is responsible for the expression of Th2 cytokines. STAT6 interacts with GATA-3 to activate the Th2 cytokine [18]. The expression of STAT6 in epithelial cells is sufficient to achieve AHR and mucus production, induced by IL-13 [19]. It is well established that cyclosporine A (CsA) has been shown to attenuate allergen-induced allergic inflammation, such as lymphocyte, eosinophil infiltration, and gene expression for IL-4 and IL-5 [20,21]. Rosiglitazone (Rosi) is an important PPARγ agonist, showing potential therapeutic effects in asthma [22]. Therefore, we used CsA and Rosi as a positive control.

Despite their diverse pharmacological activities, including anti-inflammatory activities, the molecular mechanisms of action of HD have not been fully investigated; as few reports describe the pharmacological activity of HD, further studies are needed. Moreover, there has been no in vivo research on the anti-asthmatic effects of HD, and its therapeutic mechanisms are not well known and unclear. In our study, we aimed to research the anti-asthmatic effects of HD and the involvement of the Th2/GATA-3 pathway in the effects of HD in asthmatic mice.

## 2. Results

### 2.1. Chemical Characterization of Amino Acids, Fatty Acids, Crude Protein, and Fat in HD Ethanol Extract

The main components of HD are crude protein and crude fat, which comprised 29.95% and 15.92% of the HD, respectively. As shown in Table 1 and Table 2, approximately 40 compounds were identified in the ethanol extract of HD, using an amino acid analyzer and GC-FID. Proline, glutamic acid, tyrosine, serine, glycine, alanine, valine, oleic acid, palmitoleic acid, palmitic acid, and linoleic acid were the main components of the ethanol extract of HD. The relative content of the compounds, expressed as mg/100 g, are listed in Table 1 and Table 2. Among these, proline, glutamic acid, and tyrosine were the major amino acids, and palmitic acid, palmitoleic acid, and linoleic acid were the major fatty acids.

There were 16 types of amino acids and 23 types of fatty acids in HD. Among them, seven amino acids were essential to human life. Proline was the most abundant of the 16 amino acids, and oleic acid was the most abundant of the fatty acids.

Furthermore, the HPLC analysis results (data not shown) showed chromatograms of the several compounds and the eluent was detected at 220 nm. Several common characteristic peaks were found in the fingerprint; however, the components responsible for the peaks were not identified in this study.

### 2.2. Effects of HD, CsA, and Rosiglitazone on AHR Induced by Methacholine Stimulation

**Figure 1:**
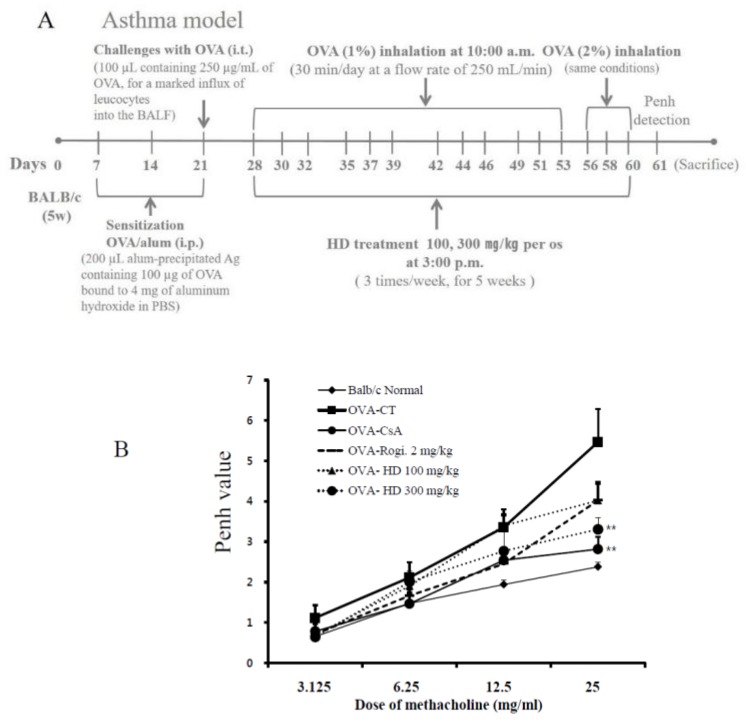

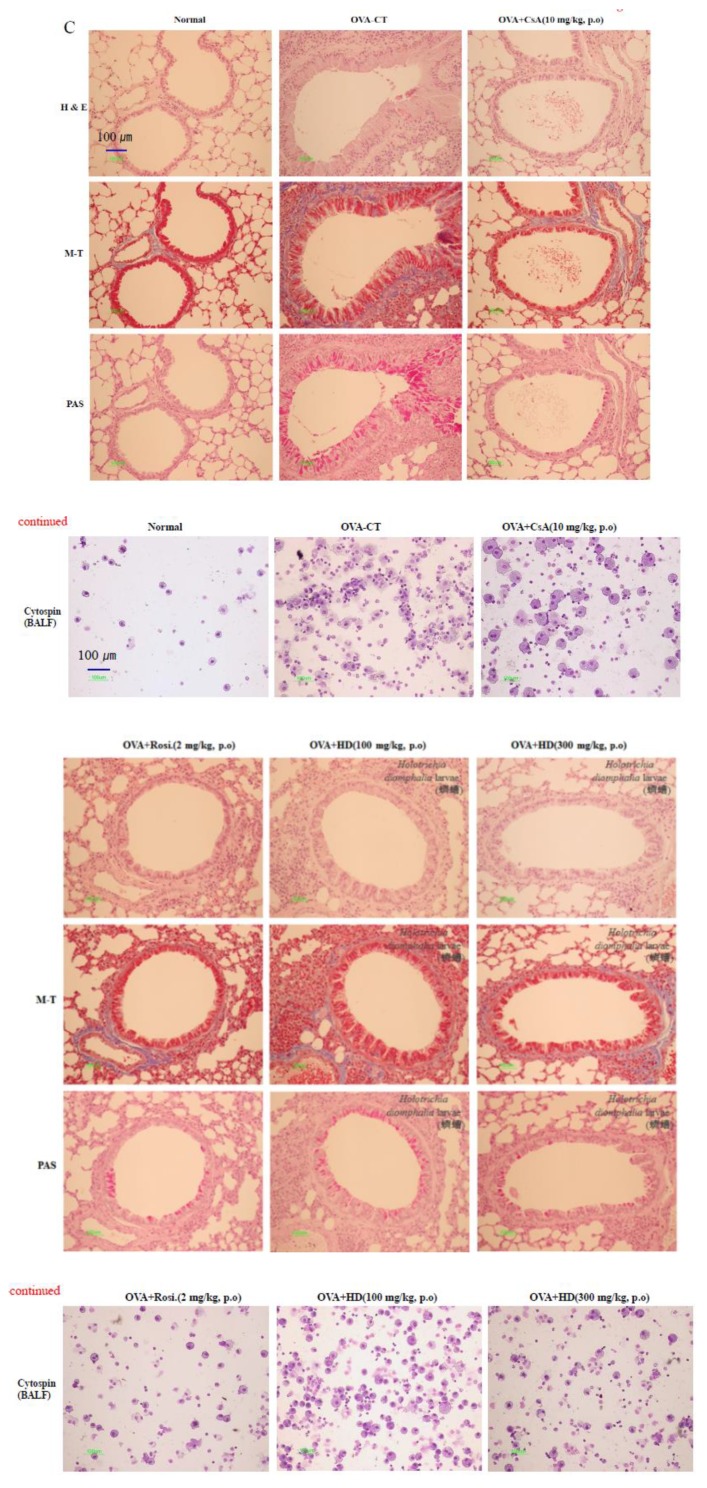
Scheme illustration of ovalbumin (OVA) sensitization and challenge protocol (**A**). The suppressive effects of HD on airway hyperresponsiveness (AHR) in OVA-challenged mice were measured using non-invasive whole-body plethysmography (**B**). Inhibitory effects of HD on histological markers of airway inflammation (H & E, M-T, and PAS staining) in lung tissue and bronchoalveolar lavage fluid (BALF) cytospin (×400 images of cytospin slide) in the OVA-induced mouse model of allergic asthma (**C**). Airway responsiveness to aerosolized methacholine was measured with a Buxco box. Data represent the mean ± SEM of 6 independent experiments. ** *p* < 0.01 for the OVA control group versus the experimental groups comparisons. N: Normal BALB/c mice; CT: OVA inhalation plus vehicle; CsA: OVA inhalation plus cyclosporine A, 10 mg/kg; Rosi: OVA inhalation plus rosiglitazone, 2 mg/kg; HD: OVA inhalation plus HD (100 or 300 mg/kg).

We evaluated the effect of HD on AHR in vivo by using the methacholine test (Penh system), which uses a gradual increase in doses of methacholine to evaluate whether HD improved the AHR in asthmatic mice. AHR induced by methacholine at concentrations of 3.125, 6.25, 12.5, and 25 mg/kg was assessed 1 day after the final OVA challenge by non-invasive whole-body plethysmography. Normal mice exhibited a weak response to methacholine, as shown in Figure 1B. However, Penh values were significantly increased in OVA-induced control mice, compared with the normal group.

Both HD (300 mg/kg) and CsA treatment significantly diminished the Penh values (*p* < 0.01). However, there was no significant changes between AHR in HD (100 mg/kg)-supplemented OVA-induced mice and OVA-induced control mice. The treatment of mice with HD (300 mg/kg) attenuated the increase in AHR, and resulted in effects similar to CsA (Figure 1B).

### 2.3. Histological Analysis of Lung Tissues

To evaluate the suppressive effects of HD on the histopathological changes in the OVA-induced mouse model of asthma, the lung tissues were stained with H & E, M-T, and PAS staining solution.

H & E staining of the lungs showed that HD and rosiglitazone diminished eosinophil infiltration in the peribronchiolar and perivascular region of the lungs in asthmatic mice, similar to the results obtained after CsA treatment (Figure 1C). M-T staining showed that collagen deposition in peribronchial and perivascular tissue was lower in HD- and rosiglitazone-treated asthmatic mice, compared to untreated asthmatic mice. We used PAS staining to investigate goblet cell hyperplasia and mucus hypersecretion in the bronchus, and demonstrated that HD, CsA, and rosiglitazone all clearly diminished goblet cell hyperplasia, compared that of OVA-sensitized mice (Figure 1C).

The number of PAS-positive goblet cells was significantly higher in the OVA-induced control group than in the normal group. Treatment with HD (100 or 300 mg/kg) resulted in a significantly lower number of PAS-stained cells. The lungs of HD- and rosiglitazone-treated OVA-induced mice showed the histopathological characteristics of light eosinophil infiltration, diminished alveolar epithelial cell hypertrophy, lower collagen deposition, and weak mucus production, compared with the control mice (Figure 1C). CsA significantly diminished perivascular and peribronchial lung inflammation.

### 2.4. HD, CsA, and Rosiglitazone Attenuate Airway Eosinophil and Inflammatory Cell Infiltration into the Lung and BALF

**Figure 2:**
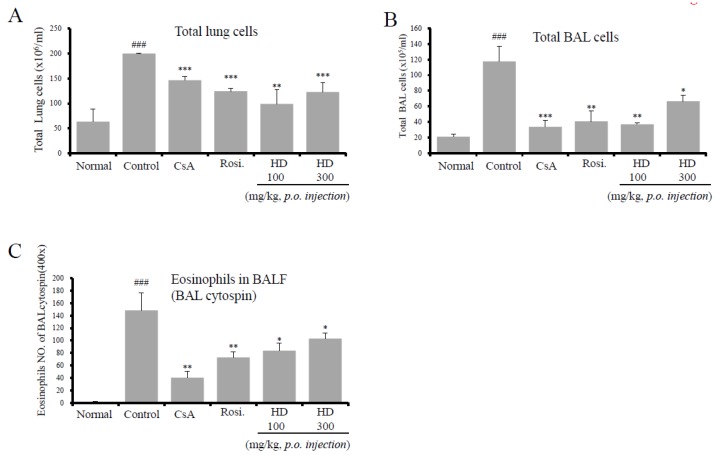
The effects of HD on total BALF cells, total lung cells, and eosinophils in BALF. Total lung cells (**A**), total BAL cells (**B**), and eosinophils in BALF (**C**). Results are expressed as mean ± SEM (*n* = 6). * *p* < 0.05, ** *p* < 0.01, and *** *p* < 0.001, for the OVA control group versus the experimental group comparisons. ^###^ *p* < 0.001, for the normal group versus the OVA control group comparison. N: Normal BALB/c mice; CT: OVA inhalation plus vehicle; CsA: OVA inhalation plus cyclosporine A, 10 mg/kg’ Rosi: OVA inhalation plus rosiglitazone, 2 mg/kg; HD: OVA inhalation plus HD (100 or 300 mg/kg).

To confirm the anti-asthmatic effect of HD in an asthmatic mouse model, the total number of lymphocytes and the number of eosinophils were determined in the lung tissues, as well as BALF. BALF was collected and Diff-Quik staining was used to calculate the total cell count and to assess the cell morphology. As shown in Figure 2, the total number of lung cells isolated from normal mice was (6.35 ± 2.55) × 10^7^, whereas a significantly higher number, (20.00 ± 0.1) × 10^7^, was isolated from the control mice. HD (20 mg/kg), rosiglitazone, and CsA reduced the OVA-induced recruitment of total lymphocytes into the lung tissues (Figure 2A).

Significant influx of inflammatory cells to BALF was observed in the OVA-challenged mice. HD (100 and 300 mg/kg) reduced the total number of BALF cells and the number of eosinophils in BALF (Figure 2B,C). Compared with (2.1 ± 0.29) × 10^5^ cells in the normal group, the total number of cells in BALF, (11.77 ± 1.99) × 10^5^, was significantly higher in the OVA-challenged control group. However, the HD-treated mice (100 and 300 mg/kg) contained (3.7 ± 0.2) × 10^5^ and (6.6 ± 0.76) × 10^5^ cells, respectively, which were significantly lower numbers of cells than in the control group. The inhibitory effect of HD was comparable with those of rosiglitazone and CsA.

### 2.5. Suppressive Effect of HD, CsA, and Rosiglitazone on the Absolute Number of Immune Cell Subsets in OVA-Induced Asthmatic Mouse Lung

To evaluate the different subpopulations of lymphocytes, and to confirm whether the increases in inflammation and AHR found in OVA-induced asthmatic mice were reflected by an altered distribution of T cell subsets, the lung cells were analyzed by flow cytometry. The effects of HD and rosiglitazone on the number of T cell subsets were investigated in normal mice, as well as in OVA-induced mice and the other experimental groups (Table 1). In OVA-induced control mice, the number of lymphocytes in the subsets of the CD3^+^/CD4^+^, CD3^+^/CD8^+^, Gr-1^+^/CD11b^+^, and CD4^+^/CD69^+^ double-positive cells in the lungs were higher than those in normal mice. A significant proportion of the CD11b^+^/Gr-1^+^ population may constitute the neutrophil population. Compared with the control group, mice in the HD-treated group had significantly lower absolute numbers of immune cell subtypes in lung tissues. HD treatment resulted in a significant decrease in the inflammatory T lymphocyte subsets in the lungs, as shown by the changes in the number of Gr-1^+^/CD11b^+^ neutrophils and CD4^+^/CD69^+^ early-activated T cells. CsA administration resulted in a significant decrease in the number of T cell subsets and Gr-1^+^/CD11b^+^ neutrophils. Furthermore, the reduction in Gr-1^+^/CD11b^+^ neutrophils was accompanied by a reduction in the number of eosinophils in BALF, the total number of lung cells, and the total number of BALF cells (Table 3, Figure 2A–C).

### 2.6. Inhibitory Effect of HD, CsA, and Rosiglitazone on Th2 Cytokines in BALF and IgE Production in Serum

OVA-induced asthmatic mice are characterized by an increase of Th2 cytokines and IL-17A.

**Figure 3:**
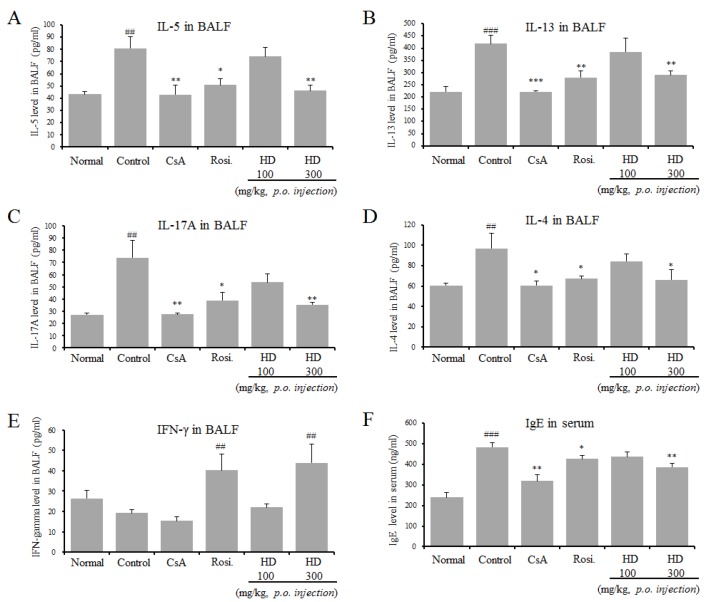
The effects of HD on Th2 cytokines (IL-4, IL-5, and IL-13), IL-17A, and IFN-γ in BALF, and the levels of total IgE in serum. IL-5 in BALF (**A**), IL-13 in BALF (**B**), IL-17A in BALF (**C**), IL-4 in BALF (**D**), IFN-γ in BALF (**E**), and IgE in serum (**F**). Each point represents the mean ± SEM values for 6 mice. * *p* < 0.05, ** *p* < 0.01, and *** *p* < 0.001, for the OVA control group versus the experimental group comparisons. ^#^ *p* < 0.05, ^##^ *p* < 0.01 and ^###^ *p* < 0.001, for the OVA control group versus the normal group comparison. N: Normal BALB/c mice; CT: OVA inhalation plus vehicle; CsA: OVA inhalation plus cyclosporine A, 10 mg/kg; Rosi.: OVA inhalation plus rosiglitazone, 2 mg/kg; HD: OVA inhalation plus HD (100 or 300 mg/kg).

To study whether HD, rosiglitazone, and CsA affected Th2 cytokine production in BALF, the levels of Th2 cytokines (IL-4, IL-5, and IL-13) were detected by ELISA after the final OVA challenge. As shown in Figure 3, IL-4, IL-5, and IL-13 levels were significantly lower in the 300 mg/kg HD-, rosiglitazone-, and CsA-treated mice, compared with those in control mice. The level of the Th1 cytokine (IFN-γ) in BALF was analyzed by ELISA, and it was found that HD (300 mg/kg) and rosiglitazone—but not CsA—increased IFN-γ production (Figure 3E). HD also significantly inhibited the levels of IL-17A. The main characteristic of the asthmatic mice is a high numerical value of IgE in serum. Therefore, the levels of IgE were investigated in the sera of OVA-induced mice and mice treated with PBS only, HD, rosiglitazone, and CsA. Total IgE levels in the sera of OVA-induced control mice were significantly higher than those in sera from normal mice. HD (300 mg/kg), rosiglitazone, and CsA significantly reduced the level of IgE (Figure 3C). Specifically, HD reduced the level of IgE in a dose-dependent manner. However, there were no significant differences in total IgE levels in BALF between the groups (data not shown).

### 2.7. Suppressive Effects of HD, CsA, and Rosiglitazone on IL-5, IL-13, IL-4, Eotaxin-2, GATA-3, and Loxl2 Gene Expression in Lung Tissue

**Figure 4:**
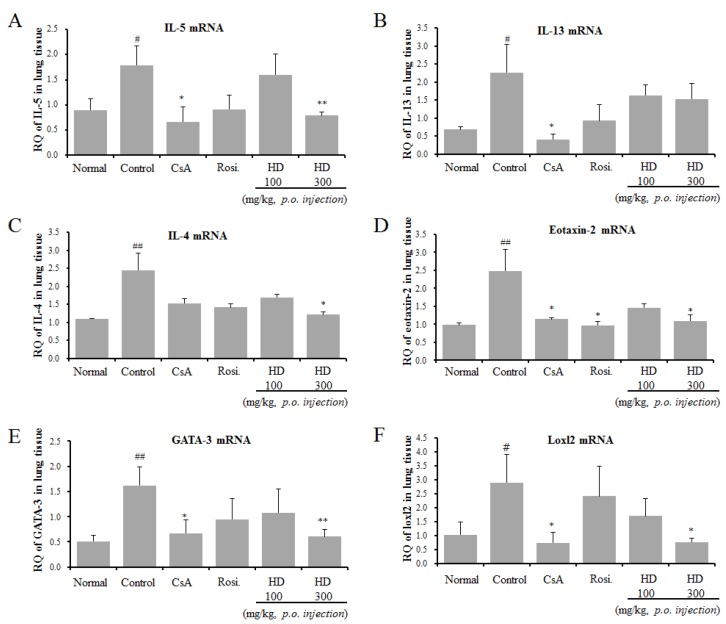
Effects of HD, CsA, and rosiglitazone on IL-5, IL-13, IL-4, eotaxin-2, GATA-3, and loxl2 mRNA expression in the lung tissue in an OVA-induced asthmatic mouse model. IL-5 mRNA (**A**), IL-13 mRNA (**B**), IL-4 mRNA (**C**), Eotaxin-2 mRNA (**D**), GATA-3 mRNA (**E**), and Loxl2 mRNA (**F**). Samples were analysed by qRT-PCR. The results are expressed as RQ to control. * *p* < 0.05 and ** *p* < 0.01, for the OVA control group versus the experimental group comparisons. ^#^ *p* < 0.05 and ^##^ *p* < 0.01, for the OVA control group versus the normal group comparison.

As the ELISA results of BALF showed that HD inhibited Th2 cytokine production, we, then, investigated the expression of mRNA for asthma-associated cytokines and mediators from OVA-induced asthmatic lung tissues by using qRT-PCR (Figure 4). In support of the ELISA results, HD treatment suppressed the mRNA expression of IL-5 and IL-4. The mRNA expression of IL-13 was also inhibited by HD, but the decrease was not significant. We examined the mechanism through which HD attenuated eosinophil recruitment into the lungs through the analysis of production of eotaxins in lung tissue. No significant effect of HD was observed on the mRNA expression of eotaxin-1 (data not shown). In contrast, HD significantly suppressed the mRNA expression of eotaxin-2 (Figure 4D). GATA-3 is a master transcription factor, regulating the development of native CD4^+^ T cells to Th2 cells. The differential expression of Th2 cytokines by HD led us to examine the mRNA expression of GATA3 in lung tissue. The results from qRT-PCR revealed that HD significantly downregulated the mRNA expression of GATA-3. However, the GATA-3 expression in rosiglitazone-treated mice was not significantly different from that in the asthmatic mice (Figure 4E). Expression of the loxl2 gene, related to airway remodeling, was higher in the asthmatic control group [23]. Our results showed that HD significantly downregulated the mRNA expression of loxl2. The above results were accompanied by changes in eosinophil influx (Gr-1^+^/CD11b^+^) (Table 3, Figure 1C, and Figure 2C), BAL cytokines (IL-4, IL-5, and IL-17A) (Figure 3), and total IgE level in serum (Figure 3F).

### 2.8. Suppressive Effects of HD, Rosiglitazone, and CsA on Intracellular ROS

The overproduction of ROS is consistent with the degree of airway hyperreactivity; moreover, excessive production of ROS results in bronchial hyperresponsiveness, which is a feature of asthma [24]. We evaluated the effects of HD, rosiglitazone, and CsA on ROS generation in lung cells.

**Figure 5:**
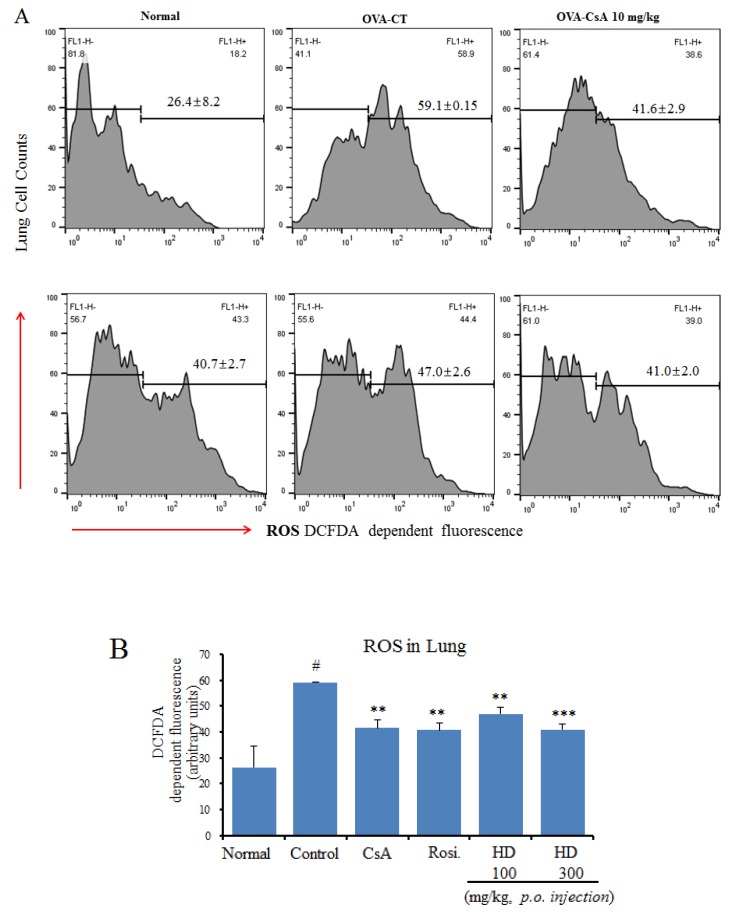
Antioxidant effects of HD, CsA, and rosiglitazone on the intracellular ROS levels in lung tissue of an asthmatic mouse model: (**A**) The generation of fluorescent oxidized DCF, shown as arithmetic mean ± SE optical density (O.D.); (**B**) Intracellular ROS level, immediately analyzed by a Cellular Reactive Oxygen Species Detection Assay Kit (ab113851, Abcam, Cambridge, MA, USA). ** *p* < 0.01 and *** *p* < 0.001, for the OVA control group versus the experimental groups comparisons. ^#^ *p* < 0.05 for the OVA control group versus the normal group comparison.

In OVA-induced asthma model mice, the level of pulmonary ROS was significantly higher than that in the normal group. However, the ROS levels were significantly lower in the HD (300 mg/kg)-treated group than in the OVA control group. The administration of HD dose-dependently suppressed OVA-induced ROS production (Figure 5A).

### 2.9. Effect of HD, CsA, and Rosiglitazone on Cytokine Expression in Vitro

**Figure 6:**
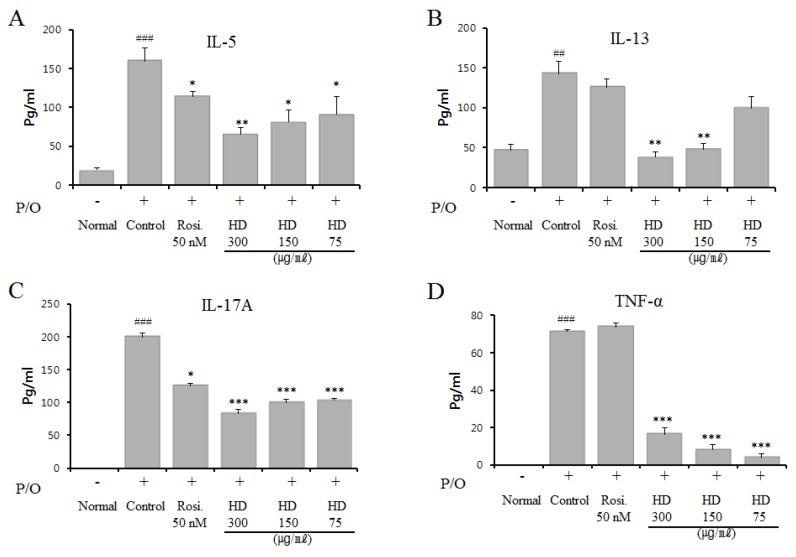
Effects of HD, CsA, and rosiglitazone on PMA- and OP-induced cytokine production of IL-5, IL-13, IL-17A, and TNF-α in EL-4 T cells. IL-5 (**A**), IL-13 (**B**), IL-17A (**C**), and TNF-α (**D**). EL-4 T cells were plated at a density of 5 × 10^5^ cells/mL in 6-well plates, stimulated with PMA (1 ng/mL) and OP (5 µM) for 1 h, and incubated with HD for 24 h. The amount of each cytokine in the supernatants was determined using ELISA kits (eBioscience, San Diego, CA, USA), following the manufacturer’s instructions. The values are presented as the mean ± SEM, for triplicate samples from each group. * *p* < 0.05, ** *p* < 0.01, and *** *p* < 0.001, for the OVA control group versus the experimental group comparisons. ^##^ *p* < 0.01 and ^###^ *p* < 0.001, for the OVA control group versus the normal group comparison.

EL-4 T cells were treated with HD at diverse concentrations, but no influence on cell viability was found (determined by the MTT assay, data not shown); the cells were stimulated with PMA and OP for 24 h. To investigate the possible effect of HD on T cell activation, we investigated the effects of HD on the expression of cytokines IL-5, IL-13, IL-17A, and TNF-α in PMA and OP-activated EL4 T cells in vitro. As shown in Figure 6, the expression of IL-5, IL-13, IL-17A, and TNF-α in the media was increased by activation with PMA/OP. When EL-4 T cells were treated with HD (75, 150, or 300 μg/mL), the secretion of IL-5, IL-13, IL-17A, and TNF-α induced by PMA/OP was significantly reduced (Figure 6).

Particularly, treatment with 300 μg/mL HD resulted in the maximum inhibition of the secretion of these cytokines (not shown). These results were similar to those for IL-5 and IL-13 in BALF and lung tissues (Figure 3 and Figure 4).

### 2.10. Effect of HD, CsA, and Rosiglitazone on the Protein Expression of Transcription Factors in EL-4 T Cells

**Figure 7:**
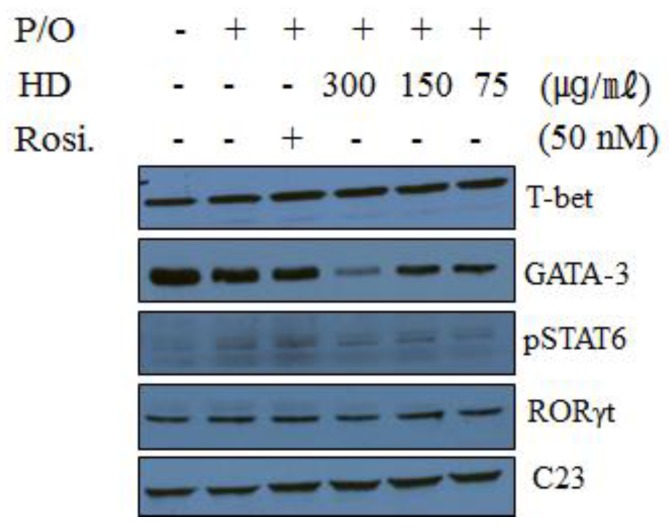
Effect of HD, CsA, and rosiglitazone on the protein levels of T-bet, GATA3, pSTAT6, and RORγt, by western blot in EL-4 T cells. EL-4 T cells were plated at a density of 5 × 10^5^ cells/mL in 6-well plates. The cells were stimulated with PMA (1 ng/mL) and OP (5 µM) for 1 h. HD was added to each plate at concentrations of 75, 150, or 300 µM. After 24 h of incubation, nuclear extracts were separated using a Nuclear/Cytosol Fractionation Kit (BioVision Incorporated, Milpitas, CA, USA), according to the manufacturer’s instructions. C23 was used as an internal control.

We performed western blotting, to investigate whether HD influenced the nuclear expression of various transcription factors known to regulate the expression of IL-4, IL-5, IL-17A, and IL-13. It is well established that Th1, Th2, and Th17 cells express distinct transcription factors such as T-bet, GATA-3, and RORγt, respectively. The expression of T-bet, GATA-3, and p-STAT6 proteins was also measured by western blot analysis of the nuclear fractions of EL-4 T cells. The analysis showed that the nuclear expression of the T-bet protein was slightly increased by HD treatment. HD treatment (300 μg/mL) significantly suppressed the nuclear expression of GATA-3; however, the expression of the p-STAT6 protein was only slightly reduced and was difficult to determine (Figure 7). HD also caused a slight suppressive effect on RORγt expression. These data suggest that the suppression of Th2 cytokines by HD may be mediated through a reduction in the GATA-3 transcription factor in EL-4 T cells. These results were similar to those of GATA-3 in lung tissue (Figure 4E).

## 3. Discussion

Asthmatic inflammation is characterized by eosinophil infiltration, airway inflammation, and the activation of Th2 cells, which secrete IL-4, IL-5, and IL-13. Histopathological changes, such as goblet cell hyperplasia, mucus secretion, airway smooth muscle hypertrophy, subepithelial fibrosis, and lung tissue infiltration of eosinophils and other immune cell subtypes are also prevalent [25]. A number of experimental models have been used to research asthma. In our study, we chose to use a mouse model of OVA-induced allergic asthma, as described in Figure 1A.

As previously mentioned in the introduction, HD reduces the production of inflammatory cytokines (IL-1 and IL-6) and exerts a range of pharmacological properties, including antioxidant, antifibrotic, antifungal, and antibacterial activities. In particular, HD may be a potent antifibrotic agent. Further investigation is needed to clearly elucidate the mechanism through which HD inhibits collagen deposition in an asthmatic mouse model. These preliminary findings indicated that HD has the potential to be used in the treatment of inflammatory diseases, such as asthma. Therefore, we hypothesized that HD may suppress inflammation of the airway and the lungs. Our results showed that both HD and CsA suppressed the level of Penh values, lymphocytes, eosinophil infiltration, mucus secretion, and lung tissue inflammation, and decreased the Th2 cytokines (IL-4, IL-5, and IL-13), IL-17A, and total IgE in BALF and sera (Table 3, Figure 1, Figure 2, Figure 3 and Figure 4). The mRNA expression of IL-5, IL-4, eotaxin-2, GATA-3, and loxl2 in lung tissues was reduced in HD-treated (300 mg/kg) mice. CsA has been shown to improve lung function and inhibit airway inflammation, such as lymphocytic and eosinophilic infiltration, and Th2 cytokine production [26], suppress IL-17 production in CD4^+^ T cells through the inhibition of PI3K/Akt and NF-kB [27], and has a well-established clinical efficacy for the improvement of lung function in severe asthma; therefore, we used CsA and rosiglitazone as positive controls. The main strategy for the treatment of asthma is the investigation of the functions of specific T cell subsets. The asthmatic model, used in the previous study, described an in vivo asthmatic mouse model with a distinct sensitization and challenge phase, characterized by OVA-induced airway inflammation and AHR dependent on CD4^+^ and CD8^+^ T cells [28]. CD4^+^ T cells and CD8^+^ T cells are well known to produce IL-4, IL-5, and IL-13 [29,30].

A substantial proportion of the Gr-1^+^/CD11b^+^ cell populations may constitute the neutrophil, eosinophil population [31]. CD69 on CD4^+^ T cells plays an important role in the development of eosinophilic airway inflammation and AHR by affecting the efficient migration of Th2 cells and eosinophils into the asthmatic lung. OVA-induced eosinophilic airway inflammation, AHR, and mucus hyperproduction were attenuated in CD69-deficient mice [32]. The upregulated numbers of activated Gr-1^+^/CD11b^+^, as well as CD4^+^ and CD8^+^ T cells, and CD4^+^/CD69^+^-positive cells in BALF and the lung of OVA-induced mice were consistent with susceptibility to lung inflammation. Moreover, these numbers of cells were significantly decreased by treatment with HD (300 and 100 mg/kg) and CsA (Table 3). These results were consistent with the reduction in the number of eosinophils in BALF (Figure 1C and Figure 2C). Th2 and Th1 cells are regulated by the transcription factors GATA-3 and T-bet, respectively [33]. IL-17A is also involved in numerous aspects of the pathogenesis of allergic asthma and airway remodeling [34]. RORγt was known to be necessary for the differentiation of Th17 cells [35], and is involved in IL-17A secretion. Further, the increased production of IL-17A functioned synergistically with IL-13 which was present during the allergic airway inflammation, increasing AHR [36]. IL-17A increased airway mucus overexpression in an asthmatic mouse model of airway inflammation [37].

GATA-3 expression was reduced in the HD-treated (300 mg/kg) group, similar to the total expression of IL-4, IL-5, IL-13, IL-17A, and IgE (Figure 3 and Figure 4); these results are similar to those reported in vitro (Figure 7). The T-bet expression in the lung tissue of mice in the HD-treated groups was not significantly altered, compared with mice in the OVA-induced control group, particularly in nuclei (data not shown). However, T-bet levels in EL-4 T cells (in vitro) were slightly increased by HD. STAT6 is a crucial factor for Th2 polarization [38] and the expression of chemokines, such as eotaxins [39]. STAT6-deficient mice do not develop eosinophilic airway inflammation or AHR, in an asthmatic mouse model of allergic airway disease [40]. STAT6 regulates the expression of GATA-3 transcription factor, which is responsible for the production of Th2 cytokines [41]. However, GATA-3 can also induce transcription of the Th2 cytokines (IL-4, IL-5, and IL-13) independent of the STAT6 pathway [42]. HD (300 mg/kg) and CsA also suppressed B cell-dependent production of IgE in serum (Figure 3F) and, therefore, we suppose that HD suppressed total IgE production through the inhibition of IL-4 and GATA-3 (Figure 3, Figure 4, and Figure 7). HD may also modulate the imbalance of the Th1/Th2-associated transcription factors, T-bet, GATA-3, and STAT6 in asthmatic mice. The expression of GATA-3 in Th2 cells was significantly suppressed after HD (300 mg/kg) treatment. Eotaxin-2, which is a major chemoattractant for eosinophils, is highly expressed in allergic inflammatory tissues [43]; it is also a featured eosinophil chemokine, likely to be involved in the IL-4-associated immune responses and allergen-induced asthma [39]. The gene expression of loxl2, related to airway remodeling, was higher in the asthma group [23]. As shown in Figure 4, we have provided the first report that HD (300 mg/kg) and CsA clearly downregulated the mRNA expression of loxl2 in the lung tissues of asthmatic model mice.

For the majority of popular herbal medicines, the therapeutic mechanisms of their pharmacological actions, as well as the identity of their active compounds, remain unknown [44]. The underlying inner therapeutic mechanisms that account for the anti-inflammatory activities and the effective chemicals of HD have remained largely unexplored. In addition, the chemical components and anti-asthmatic mechanisms of HD have not previously been studied in depth. Therefore, a principal ingredient analysis was performed to determine the chemical constituents of the extract, and identify the main chemical components through amino acid and fatty acid analysis. As described in the results, the main components of HD are crude protein and crude fat, present at 29.95% and 15.92%, respectively. In total, as shown in Table 1 and Table 2, approximately 40 compounds were identified in the ethanol extract of HD by using amino acid analysis and GC-FID. Oleic acid, palmitoleic acid, palmitic acid, linoleic acid, proline, glutamic acid, tyrosine, serine, glycine, alanine, and valine were the main components of the ethanol extract of HD; oleic acid was the most abundant fatty acid and proline was the most abundant of the sixteen amino acids. The relative content (mg/100 g) of each compound is listed in Table 1 and Table 2. Recently, evidence has been provided about the potent use of a mixture of fatty acids, in which the main components were oleic, linoleic, palmitic, and linolenic acids, for the treatment of cutaneous inflammatory and allergic disorders [9]. Fatty acids regulate immune responses, and it has been proved that they influence the incidence of IgE-mediated allergic disorders. Maternal palmitic acid, α-linolenic acid, and total *n*-3 polyunsaturated fatty acid (PUFA) intake may reduce the risk of asthma in offspring [45]. According to previous results, the sufficient free fatty acids (palmitoleic, palmitic, oleic, and stearic acids) are important in inducing a large reduction in the apparent heat resistance of bacterial spores [46]. Eight fatty acids (palmitoleic acid, oleic acid, linoleic acid, lauric acid, myristic acid, palmitic acid, stearic acid, and elaidic acid), which are contained in the maternal fluids, produced anxiolytic-like effects in adult rats [47].

Pharmacological effects on the allergic diseases induced by the main components of the ethanol extract of HD have been studied individually. Oleic acid reduced IgE binding to allergens [48], linoleic acid exerted modest anti-inflammatory effects in subjects with allergies [49] and reduced airway inflammation, AHR, BAL inflammatory cell count, and lung IL-5 expression in an asthmatic mouse model of allergic asthma through a PPARγ-dependent mechanism [50]. Several direct links between those major compounds and the inhibition of the GATA-3/Th2 responses have been reported; for example, *Camellia japonica* oil suppressed asthma occurrence through the GATA-3, IL-4, and IL-5 pathways and its effective and major component is oleic acid [51]. A high-fat diet enriched with oleic acid impairs contact AHR response to 2,4,6-trinitrochlorobenzene (TNCB) and FITC in susceptible mice by inducing a lack of the IL-4 signal [52]. Kelly et al. reported that linoleic acid decreased IL-4 secretion by splenocytes, compared to those in the control group [53]. Moreover, immunoreceptor tyrosine-based inhibitory motifs have a physiologic negative regulatory role in signaling through IL-4Rα, especially by IL-13 [54]. Poly-γ-glutamic acid (γ-PGA) alleviates pathologic symptoms in a Th2-biased asthmatic model, in which Th2 polarization plays a crucial role [55].

A positive correlation was also found between the intake of palmitoleic and oleic acids and allergic sensitization [56]. Palmitoleic acid has anti-inflammatory activities, which were linked to increased PPAR expression, an inhibitor of NFκB [57]. In contrast, palmitic acid increased the number of lung macrophages and augmented airway inflammation in a mouse model [58]. It has been supposed that amino acids contribute to numerous antioxidant and immunological effects relevant to allergic asthma pathogenesis. However, the roles of amino acids and their mixtures in asthma models and allergic disorders have not been fully investigated. In particular, glutamic acid, glycine, and methionine, which collectively contribute to glutathione metabolism, have emerged as critical antioxidants, which may affect susceptibility to allergic asthma, whereas arginine and phenylalanine may produce adverse effects in asthma [59]. Poly-γ-glutamic acid inhibited allergic lung inflammation through the Toll-like receptor-4-dependent pathway in a mouse model of asthma [55], and glutamic acid has been recommended for asthma patients [60]. However, increased nitrotyrosine, a marker of protein tyrosine, was identified in the airway epithelial cells and inflammatory lung cells. It was also correlated with increased exhaled iNOS, NO, AHR, and eosinophilic inflammation [61]. l-proline plays an important role in the pathways of fibrosis, by contributing to collagen deposition [62]. In insects, the fatty acid concentrations are sufficient to affect human health. In our study, we reported the characterization of fatty acids and amino acids from a coleopteran insect, HD. In a meaningful and comparable result, Seo et al. reported that palmitic acid and methionine were the most common metabolites, as potential biomarkers in the plasma samples of OVA-induced asthmatic mice [63]. The complex and diverse pharmacological profiles of these components, as well as their individual anti-inflammatory and anti-asthmatic properties, suggest that these ingredients in HD may be involved in the antiasthmatic effects observed in our results.

ROS may augment and initiate inflammation, but can also result from inflammation. ROS, which is well known to contribute to oxidative stress, is predominantly produced by inflammatory cells and eosinophils recruited to the airway and lung in asthma [64]. Airway oxidative stress by ROS may affect systemic allergic inflammation through the increase of the Th17 immune responses [65]. IL-17A production in lung cells was suppressed in HD-treated (300 mg/kg) mice, and its production in the HD-treated groups (Figure 6) in vitro was decreased. It is possible that HD can inhibit IL-17A production through the inhibition of ROS and RORγt, main transcription factors for Th17 cells. Recent investigations have shown that *Holotrichia parallela* (Motschulsky) reduced oxidative stress and possessed confident antioxidant activities, in addition to its anti-inflammatory activities [66]. In our results, intracellular ROS production was at a significantly lower level in the HD-treated group than in the OVA-induced control group (Figure 5). These results indicate that HD treatment has a protective effect against oxidative stress through the significant decrease in ROS.

The results also indicated that HD exerts inhibitory effects on airway inflammation, and that this effect was induced by the inhibition of Th2 cytokines (IL-5, IL-13), IL-17A, and total IgE through the GATA-3, pSTAT6, and RORγt transcription pathways. It is unclear why there is a difference in the responsive to CsA, rosiglitazone, and HD among these factors. One of the possibilities is that the differential activities of CsA, rosiglitazone, and HD on cytokines and transcription factors may be involved in the overall response. Based on previous results and our preliminary investigation (data not shown), which was conducted to demonstrate the safety of this compound, effective and safe concentrations were used in our study. Therefore, it can be suggested that HD is a more effective candidate for a traditional medicine with fewer side effects, based on our results and numerous other reports.

## 4. Materials and Methods

### 4.1. Preparation of Ethanol Crude Extracts and Other Reagents

The samples of HD were purchased from Omniherb Korean Herbs Co. Ltd. (Daegu, Korea) in May 2016, and identified by Professor Young-Cheol Lee, College of Korean Medicine, Sangji University in Wonju, Korea. Voucher specimens (no. 2016-SJHD-1) were deposited in our laboratory at the Department of Herbology, College of Korean Medicine, Sangji University Wonju 220-702, Republic of Korea. Dried and chopped HD (1 kg) was refluxed three times with 70% ethanol for 8 h. The materials were filtered under reduced pressure at 40 °C by using a vacuum rotatory evaporator (BUCHI B-480, Flawil, Switzerland) and dried in a freeze-drier (EYELA FDU-540, Tokyo, Japan) to yield the HD extract (114.57 g). The yield (*w*/*w*) of the extract was approximately 11.46%. OVA, methacholine, and aluminum hydroxide were purchased from Sigma. All other chemical reagents were analytical grade and were purchased from Sigma, unless otherwise indicated.

### 4.2. Amino Acids and Total Crude Protein Analysis

A previously described analysis method with minor modifications was used for measurement of amino acid content and total protein content of the samples [67]. In brief, 150 μg aliquots of the extract were dissolved in 100 mM HCl (75 μL). Acid hydrolysis of the sample was performed in 5 M HCl under vacuum for 24 h at 108 °C. The hydrolyzed samples were dissolved in 100 mM HCl (70 μL), and 50 μL of the solution was injected into an amino acid analyzer (Hitachi L8900, Tokyo, Japan). The amino acid composition of the sample was measured in triplicate. The total content of crude protein in the HD was analyzed by using a Kjeltec 8400 (Foss, Sweden) and calculated from the summation of the quantities of the individual amino acids.

### 4.3. Fatty Acids and Total Crude Fat Analysis

Fatty acids and the total fat content of HD were analyzed by a previously described method [68]. Briefly, for fatty acid identification and quantitation by gas chromatography-flame ionization detection (GC-FID), the sample was subjected to transmethylation/methylation procedures under sequential alkaline and acid conditions. Fatty acid methyl esters (FAME) were analyzed by using capillary gas chromatography under conditions as previously described. The result peaks were identified and compared to a commercial Certified Reference Material (FAME mix, C4:0 to C24:0, purchased from Sigma Aldrich Korea (Seoul, Korea) and quantified by area normalization. The total crude fat content of the samples was analyzed using diethyl ether by the Randall method (also known as the Soxtec method), and calculated from the summation of the individual fatty acids.

### 4.4. Animals

Five-week-old female BALB/c mice, weighing 20–25 g, were obtained from Daehan Biolink Co. Ltd. (Eumsung, Republic of Korea) and maintained in specific pathogen-free conditions at our animal breeding facilities. All animal procedures and experiments were performed in accordance with the guidelines of the Institutional Animal Care and Use Committee of Daejeon University, Republic of Korea.

### 4.5. OVA Sensitization, Inhalation, Challenge, and Enhanced Pause (Penh) Measurement

OVA preparation, sensitization, challenge, inhalation, and Penh measurements were accomplished as previously described, with minor modifications (Figure 1A) [69,70].

Mice were sensitized on three different days by intraperitoneal (i.p.) injections of 200 μL alum-precipitated antigen, containing 100 μg OVA bound to 4 mg aluminum hydroxide in PBS. They were then administered intratracheal injections of 100 μL, containing 250 μg/mL of OVA in PBS, 21 days after the first sensitization. On day 28, on the back of the tongue, the mice were exposed to aerosolized OVA for 30 min/day, 3 days/week, for 5 weeks, as previously described in Figure 1A. HD (100 and 300 mg/kg), cyclosporine A (CsA, 10 mg/kg), and rosiglitazone (2 mg/kg) were orally administered three times per week, for 5 weeks. One day after the final OVA exposure, Penh values were determined and BALF, lung cells, and serum were collected for molecular analyses.

Penh value is equal to Pause × PEF/PIF, where Pause = (Te − Tr)/Tr; PIF is peak inspiratory flow; PEF is peak expiratory flow; Te is expiratory time; and Tr is relaxation time. At 24 h after the final inhalation, the mice were placed in a plethysmographic chamber and aerosolized methacholine in increasing concentrations (3.125, 6.25, 12.5, or 25 mg/mL) was nebulized through an inlet of the chamber. Airway responsiveness was then monitored for 30 min. The differences in Penh values among groups were calculated by using an unpaired Student’s *t*-test.

### 4.6. Bronchoalveolar Lavage Fluid (BALF)

As previously described [70], after Penh measurement, the mice were anesthetized with an i.p. injection of sodium pentobarbitone (100 mg/kg body weight) in phosphate-buffered saline (PBS). The trachea was cannulated and BALF was collected by lavaging the airway lamina. Briefly, the BAL cells in the lungs were recovered by flushing three times with 1 mL PBS (1 mM ethylenediaminetetraacetic acid (EDTA), 10% fetal bovine serum (FBS)) into the lungs through the trachea. The total cell count was determined using a hemocytometer, and 100 µL of fluid was applied onto glass slides by use of a cytospin centrifuge (400× *g*, 4 min). Differential cell counts in BALF were assessed after staining with a Diff-Quik Stain Set (Baxter Healthcare Corp., Miami, FL, USA). The remaining BALF supernatants were stored at −25 °C for further assessments.

### 4.7. Digestion of Lung Tissue And Cell Preparation

Lung cell suspensions were isolated by mechanical disruption and collagenase digestion. Briefly, the extracted right lobes of the lungs were minced by using sterile scalpels, followed by incubation in PBS containing 1 mg/mL collagenase IV and 2 mg/mL dispase for 30 min at 37 °C in a sterile polypropylene tube. After incubation, the lung tissues were pipetted up and down vigorously, and then filtered through a 70-µm cell strainer. The remaining lobes of the left lung were stored for mRNA extraction (upper lobe) and histopathological analyses (bottom lobe).

Between 2–4 × 10^3^ cells were transferred to glass slides (Cytospin centrifuge, 400× *g*, 4 min). The cells were counted manually, using a hemocytometer. Differential counts were performed in accordance with standard morphological criteria, as in the previously described protocol with minor modifications [70].

### 4.8. Hematoxylin-Eosin (H & E), Masson-Trichrome (M-T), and Periodic acid-Schiff (PAS) Staining

One day after the final OVA exposure, the mice were sacrificed and the lungs were harvested and inspected with histopathological analysis as in the previously described method, with minor modifications [70].

Briefly, the lung tissues were fixed with formalin, embedded in paraffin, and cut into 3-µm sections. Sections were stained with H & E and the inflammatory lymphocytic cells infiltration into lung tissues was measured by using optical microscopic investigation of the H & E-stained sections. Other sections were stained with M-T to determine collagen deposition, or with PAS to quantify mucus secretion and goblet cells in the lungs. Frozen sections of each lung tissue sample were prepared. The lung tissue was subsequently mounted and covered with Dako-mounting medium (Dako Cytomation; Carpinteria, CA, USA) and a coverslip.

### 4.9. Fluorescence-Activated Cell Sorting (FACS) Analysis

All antibodies (anti-cluster of differentiation (CD)3, CD4, CD8, CD11b, Gr-1, and CD69) for FACS were purchased from Becton Dickinson (BD) PharMingen (San Diego, CA, USA). Briefly, the cells (5 × 10^5^) were washed with FACS staining buffer. Then, the cells from lung tissues were stained with the indicated antibodies in FACS staining buffer (PBS containing 1% FBS and 0.01% NaN_3_) for 10 min on ice, and then analyzed by two-color flow cytometry using a FACSCalibur device and CellQuest software (BD Biosciences, Mountain View, CA, USA).

### 4.10. Enzyme-Linked Immunosorbent Assay (ELISA)

The expression of interleukins, such as IL-4, IL-5, IL-13, IL-17A, and IFN-γ, in BALF and total IgE in serum, were detected by using mouse interleukin ELISA kits, in accordance with the manufacturer’s instructions (R & D Systems, Minneapolis, MN, USA). All data represent the mean and standard deviation from a minimum of three separate experiments and were compared by analysis of variance (ANOVA).

### 4.11. Quantitative Real-Time PCR (qRT-PCR) in Vivo

Total RNA was extracted from the lung by RNA TRIzol reagent (Invitrogen) and cDNA was synthesized from an equal amount of RNA (3 µg per reaction) using a ReverTraAce-a-cDNA Synthesis kit (Toyobo, Tokyo, Japan), in accordance with the manufacturer’s instructions. qRT-PCR was performed using the 7500 Fast Real-Time PCR system (Applied Biosystems, Foster, CA, USA) and the following TaqMan probes and primers: GATA-3 (Mm00484683, FAM dye-labelled, ABI), LOXL2 (Mm00804740, FAM dye-labelled, ABI), and glyceraldehyde-3-phosphate dehydrogenase (GAPDH) (pn, 4352339E, VIC dye-labelled, ABI) were selected by using Assays-on-Demand Gene Expression Products (ABI). GAPDH (ABI) gene expression was used as an endogenous control. The following primers were used for analysis: mouse IL-4, sense 5′-GGATGTAACGACAGCCCTCT-3′, and antisense 5′ -GTGTTCCTTGTTGCCGTAAG-3′; IL-5, sense 5′-AGCACAGTGGTGAAAGAGACCTT-3′, and antisense 5′-TCCAATGCATAGCTGGTGATTT-3′; IL-13, sense 5′-CAGTTGCAATGCCATCCACA-3′, and antisense 5′-AGCCACATCCGAGGCCTTT-3′; eotaxin-2, sense 5′-CTGTGACCATCCCCT-CATCT-3′, and antisense 5′-CTTATGGCCCTTCTTGGTGA-3′; and β-actin, sense 5′-TCATGAAGTGTGACGTTGACATCCGT-3′, and antisense 5′-CTAGAAGCATTTGCGGTGCACGATG-3′; β-actin was used as the internal standard; and the analysis procedure used SYBR Green PCR Mastermix (ABI). The following PCR parameters were used: 2 min at 50 °C; 10 min at 94 °C; followed by 35 cycles of 1 min at 94 °C, 1 min at 60 °C, and 1 min at 72 °C. The cycle number at which the emission intensity of the sample rose above the baseline was referred to as the RQ (relative quantification), and was proportional to the target concentration. qRT-PCR analyses were performed in triplicate and in accordance with the manufacturer’s instruction.

### 4.12. Intracellular Reactive Oxygen Species (ROS) Measurement

ROS was determined by using a 2′,7′-dichlorodihydrofluorescein diacetate (DCFDA) Cellular ROS Detection Assay Kit (ab113851, Abcam, Cambridge, MA, USA), in accordance with the manufacturer’s protocol, as previously described [69]. Lung cells were washed in DCFDA solution and stained with DCFDA solution (Sigma-Aldrich Korea, Seoul, Korea) at a final DCFDA concentration of 25 μM lung cells, incubated at 37 °C for 45 min in the dark, and washed twice in DCFDA solution. The DCFDA-loaded lung cells were resuspended in 100 μL DCFDA solution and the ROS-dependent fluorescence intensity was measured at an excitation wavelength of 485 nm and an emission wavelength of 535 nm on a FACSCalibur device by using CellQuest software (BD Biosciences, Mountain View, CA, USA). The intracellular ROS expression was reported in relative fluorescence units.

### 4.13. Detection of Cytokines by ELISA and Immunoblotting Analysis in Vitro

EL-4 T cells were plated in 6-well plates at a density of 5 × 10^5^ cells/mL. The cells were stimulated with phorbol 12-myristate 13-acetate (PMA, 1 ng/mL), 4-tertoctylphenol (OP, 5 μM), and rosiglitazone (50 nM) for 1 h. HD was added to each plate at concentrations of 300, 150, or 75 μg/mL. After incubation for 24 h, the supernatants from the culture were collected and stored at −80 °C until they were used for the measurement of cytokine production. The cytokine production in the supernatants was assessed by using ELISA kits (eBioscience, San Diego, CA, USA), in accordance with the manufacturer’s instructions. The nuclear lysates were separated by using a Nuclear Fractionation Kit (BioVision Incorporated, Milpitas, CA, USA), in accordance with the manufacturer’s instructions. In brief, the nuclear lysates were separated using 10% sodium dodecyl sulfate polyacrylamide gel electrophoresis (SDS-PAGE) and the electrophoresed proteins were transferred to nitrocellulose membranes. Nonspecific binding to the membrane was inhibited by incubation of the membranes with TBST (50 mM Tris-HCl, pH 7.5, 150 mM NaCl, and 0.1% Tween-20) containing 5% fat-free milk for 1 h at RT. The membranes were incubated overnight with specific antibodies against T-bet, GATA-3, pSTAT6, and C23 (Santa Cruz Biotechnology, Santa Cruz, CA, USA) at 4 °C. Immunoreactive bands were determined by incubation of the membranes with horseradish peroxidase (HRP)-conjugated secondary antibodies and enhanced chemiluminescence reagents, as previously described [70].

### 4.14. Statistical Analysis

The data were analyzed by using an unpaired Student’s *t*-test, or ANOVA followed by Dunnett’s multiple comparison test (SPSS analysis software, version 14.0, IBM, New York, NY, USA). Statistical significance is indexed in the figures and tables to emphasize the significance in the differences between the HD-, CsA-, and rosiglitazone-treated experimental groups and the OVA-induced control group. The results were presented as the mean ± standard error of the mean, and were considered statistically significant at *p* values of <0.05 (*), <0.01 (**), or <0.001 (***), for the experimental groups versus the OVA control group comparisons and at *p* values of <0.05 (^#^), <0.01 (^##^), or <0.001 (^###^), for the normal group versus the OVA-induced control group comparisons.

## 5. Conclusions

In conclusion, our study is the first report to show that HD significantly suppressed OVA-induced lung inflammation, lymphocytes, eosinophils infiltration, and AHR in an asthmatic mouse model of allergic asthma by the inhibition of Th2 cytokines (IL-5 and IL-13) by the GATA-3 (high) and pSTAT6 (low) transcription pathways. These results suggest that HD might be a novel natural product from an insect resource showing potential as a therapeutic agent for the treatment of allergic asthma.

## Figures and Tables

**Table 1:** Composition of amino acid and total crude protein in *Holotrichia diomphalia* larvae (HD) (mg/100g).

Compounds (Amino Acid)	Formula	Result (mg/100g)
Aspartic acid	C_4_H_7_NO_4_	962.9
Threonine	C_4_H_9_NO_3_	806.4
Serine	C_3_H_7_NO_3_	1731.3
Glutamic acid	C_5_H_9_NO_4_	3210.3
Proline	C_5_H_9_NO_2_	5375.1
Glycine	C_2_H_5_NO_2_	1771.1
Alanine	C_3_H_7_NO_2_	1675.5
Valine	C_5_H_11_NO_2_	1241.5
Methionine	C_5_H_11_NO_2_S	300.9
Isoleucine	C_6_H_13_NO_2_	739.2
Leucine	C_6_H_13_NO_2_	806.5
Tyrosine	C_9_H_11_NO_3_	4009.2
Phenylalanine	C_9_H_11_NO_2_	873.8
Lysine	C_6_H_14_N_2_O_2_	855.4
Histidine	C_6_H_9_N_3_O_2_	579.9
Arginine	C_6_H_14_N_4_O_2_	786.6
Total crude protein (%)		29.95

**Table 2:** Composition of fatty acid and total crude fat in HD (g/100g).

Compounds (Fatty Acid)	Formula	Result (g/100g)
Lauric acid	C_12_H_24_O_2_	0.005
Tridecanoic acid	C_13_H_26_O_2_	0.003
Myristic acid	C_14_H_28_O_2_	0.180
Myristoleic acid	C_14_H_26_O_2_	0.026
Pentadecanoic acid	C_15_H_30_O_2_	0.046
Pentadecenoic acid	C_15_H_28_O_2_	0.001
Palmitic acid	C_16_H_32_O_2_	3.206
Palmitoleic acid	C_16_H_30_O_2_	1.792
Margaric acid	C_17_H_34_O_2_	0.069
Heptadecenoic acid	C_17_H_34_O_2_	ND
Stearic acid	C_18_H_36_O_2_	0.349
Oleic acid	C_18_H_34_O_2_	0.046 (trans)
		8.893 (cis)
Linoleic acid	C_18_H_32_O_2_	0.059 (trans)
		1.076 (cis)
Arachidic acid	C_20_H_40_O_2_	0.078
γ-Linolenic acid	C_20_H_34_O_2_	ND
Linolenic acid	C_18_H_30_O_2_	0.172
Gadoleic acid	C_20_H_38_O_2_	0.026
Heneicosanoic acid	C_21_H_42_O_2_	ND
Eicosadienoic acid	C_20_H_36_O_2_	0.009
Behenic acid	C_21_H_43_COOH	0.013
Dihomo-gamma-linolenic acid	C_20_H_34_O_2_	0.010
Erucic acid	C_22_H_42_O_2_	0.006
Eicosatrienoic acid	C_20_H_34_O_2_	ND
Arachidoninc acid	C_20_H_32_O_2_	0.171
Tricosanoic acid	C_23_H_46_O_2_	ND
Brassic acid	C_22_H_40_O_2_	ND
Lignoceric acid	C_24_H_48_O_2_	0.004
EPA	C_20_H_30_O_2_	0.060
Nervonic acid	C_24_H_46_O_2_	ND
DHA	C_22_H_32_O_2_	0.005
Total crude fat (%)		15.92

**Table 3:** Fluorescence-activated cell sorting analysis (FACS) of immune cell subsets in lung. Each point represents the mean ± SEM values for 6 mice. * *p* < 0.05, ** *p* < 0.01, and *** *p* < 0.001, for the OVA control group versus the experimental group comparisons. N: Normal BALB/c mice; CT: OVA inhalation plus vehicle; CsA: OVA inhalation plus cyclosporine A, 10 mg/kg; Rosi: OVA inhalation plus rosiglitazone, 2 mg/kg; HD: OVA inhalation plus HD (100 or 300 mg/kg).

Cell Phenotypes in Lung		Normal BALB/c	Ovalbumin-Induced Asthma Mice (Absolute No.)				
			Control	Cyclosporin A	Rosi. (2 mg/kg)	HD (100 mg/kg)	HD (300 mg/kg)
CD3^+^/CD4^+^ (×10^5^ cells)		184.30 ± 83.61	633.00 ± 10.17	351.40 ± 85.95 **	374.60± 12.82 ***	288.60± 65.99 **	350.60 ± 46.97 **
CD3^+^/CD8^+^ (×10^5^ cells)		54.60± 30.83	201.90 ± 1.09	140.70 ± 22.55 *	123.20 ± 11.27 ***	117.40 ± 29.85 *	133.70 ± 33.87 *
Gr-1^+^/CD11b^+^ (×10^4^ cells)	Lung	72.80 ± 28.69	228.00 ± 3.14	150.40 ± 37.44 *	337.70 ± 49.72	124.10 ± 28.21 **	185.10 ± 5.18 ***
CD4^+^/CD69^+^ (×10^5^ cells)		1.70 ± 1.39	20.30 ± 0.80	39.50 ± 15.98	12.10± 0.72 ***	10.60± 3.77 **	12.10 ± 2.63 **
